# Like mother like daughter: northern elephant seals exhibit fine-scale philopatry

**DOI:** 10.1007/s00442-025-05846-6

**Published:** 2025-12-10

**Authors:** Isabella G. P. Garfield, Danial G. Palance, Max F. Czapanskiy, Daniel P. Costa, Roxanne S. Beltran

**Affiliations:** 1 Department of Ecology and Evolutionary Biology, University of California Santa Cruz, 130 McAllister Way, Santa Cruz, CA 95060 USA; 2 Institute of Marine Sciences, University of California Santa Cruz, 115 McAllister Way, Santa Cruz, CA 95060 USA

**Keywords:** Natal philopatry, Pupping, Site fidelity, Pinniped, Breeding sites, Fine-scale philopatry, Natal straying

## Abstract

Natal philopatry, the practice of a mother reproducing in the same region as her natal site, has been documented in numerous species. Studies on marine mammal philopatry have primarily focused on cross-colony scales, leaving a knowledge gap for fine-scale philopatry within colonies. We sought to identify the rate of fine-scale natal philopatry in northern elephant seals, sustained site fidelity across years, and additional drivers of site selection during the breeding season. Using 20 years of mark-recapture data collected from the ~3200-m long Año Nuevo colony in northern California, we discovered high rates of fine-scale philopatry, with females pupping an average of 395 m from where they were born. Females producing pups showed high site fidelity to the site of their first pup production, although the correlation was not as strong as to their natal site. Our results have implications for within-colony genetic connectivity and generational shifts in breeding sites influenced by coastal erosion.

**Supplementary Information:**

The online version contains supplementary material available at 10.1007/s00442-025-05846-6.

## Introduction

Natal site fidelity, or philopatry, refers to the event of returning to one’s birth site to breed, and is a life history adaptation adopted by numerous species, including salmon (Hendry and Stearns 2003), sparrows (Wheelwright and Mauck 1998), seabirds (Coulson 2016; Pyle and Nettleship 2001; Spear et al. 1998), sea turtles (Stiebens et al. 2013), and pinnipeds (Fabiani et al. 2006; Hastings et al. 2017; Hoffman and Forcada 2012; Pomeroy et al. 2001; Wolf and Trillmich 2007), among others (Clutton-Brock and Lukas 2012). Wide-ranging populations benefit from philopatry because it increases the ability to locate a mate and facilitates familiarity with dynamic environmental conditions at the natal site (Clutton-Brock and Lukas 2012; Hendry and Stearns 2003). However, there is a risk of inbreeding if a population exhibits high philopatry (Coulson 2016; Clutton-Brock and Lukas 2012). Thus, a certain level of straying beyond the birth colony is expected and beneficial, as it increases the genetic diversity between colonies (Bonnell and Selander 1974). Genetic diversity is often discussed when addressing natal philopatry, either as an indicator (Stiebens et al. 2013), or as a consequence of philopatry (Clutton-Brock and Lukas 2012; Fabiani et al. 2003). While broad-scale philopatry to individual colonies has been documented in northern elephant seals (*Mirounga angustirostris*) (Condit et al. 2023; Zeno et al. 2021), fine-scale philopatry within colonies (e.g., the distance *within a colony* between a female’s natal site and her subsequent pupping site) has not been examined. Accordingly, our study aims to identify the rate of fine-scale (within-colony) natal philopatry and additional site selection drivers, including site fidelity across breeding seasons and natal straying.

The relationship between genetic variation and philopatry is exceptionally important in the case of northern elephant seals due to the low genetic variation found in the current population (Bonnell and Selander 1974; Hoelzel et al. 1993, 2024). Northern elephant seals were nearly hunted to extinction during the nineteenth century before protections were in place, resulting in an extreme genetic bottleneck in the surviving population of roughly 30 individuals (Hoelzel et al. 1993). Despite this dramatic culling, the northern elephant seal population has recovered and established over a dozen breeding sites ranging along the North American West coast from Baja California to Vancouver’s Race Rocks marine protected area, with a total estimated population size of 210,000–239,000 individuals as of 2010 (Lowry 2014; Riedman 1990). While the low genetic diversity of the species is linked to the historic population bottleneck (Hoelzel et al. 2024), high rates of philopatry could also inhibit genetic diversification (Lopes et al. 2015).

Northern elephant seals rely on colony sites for yearly breeding and molting (Riedman 1990). Adult females return to established colonies between December and February to pup. Female elephant seals prefer sandy substrate during the breeding season (Arias-del-Razo et al. 2016; Campagna and Lewis 1992). Nursing females remain with their pups for approximately 4 weeks before abruptly weaning them to resume foraging and rebuild the energy reserves lost while on the beach (Costa et al. 1986). Females return to their colonies between April and June to catastrophically molt, a haul-out period lasting 32 days on average (Worthy et al. 1992; Beltran et al. 2024). Following the molt, females undergo an 8-month foraging migration to rebuild fat reserves for the subsequent breeding season (Robinson et al. 2012). During this trip, seals travel nearly 10,000 km into the North Pacific Ocean and return only days before giving birth to their pups (Le Boeuf et al. 2000; Beltran et al. 2022; Stewart and DeLong 1995).

Our study focused on the mainland colony of northern elephant seals located at Año Nuevo Reserve, California, USA. We characterized fine-scale site selection trends using these seals as a model system by considering fine-scale natal philopatry, site fidelity, and natal straying. Our goal was to illuminate possible drivers of the broad-scale dispersal of elephant seals by distinguishing fine-scale dispersal trends. Our first objective was to assess how far mothers gave birth from their natal site within the Año Nuevo colony by mapping these distances to distinguish straying trends. We hypothesized that reduced fine-scale philopatry may be favored because it would reduce inbreeding and competition with kin, and increase genetic variation at a fine scale. Together, these factors would be beneficial for elephant seals because they are still recovering from low genetic variation from the historic population bottleneck. Our second objective was to understand whether fine-scale site fidelity was maintained across breeding seasons and years between pupping events at different temporal scales (lag years). We hypothesized that the distance between pupping sites would increase with lag years, as seals may explore new birth sites throughout their lifetimes. We also tracked the directions of natal straying to determine if regions within the Año Nuevo colony had different natal straying patterns, and compared our findings to site-selection trends observed across other northern elephant seal colonies and within other phocid species.

## Methods

### Site

**Fig. 1:**
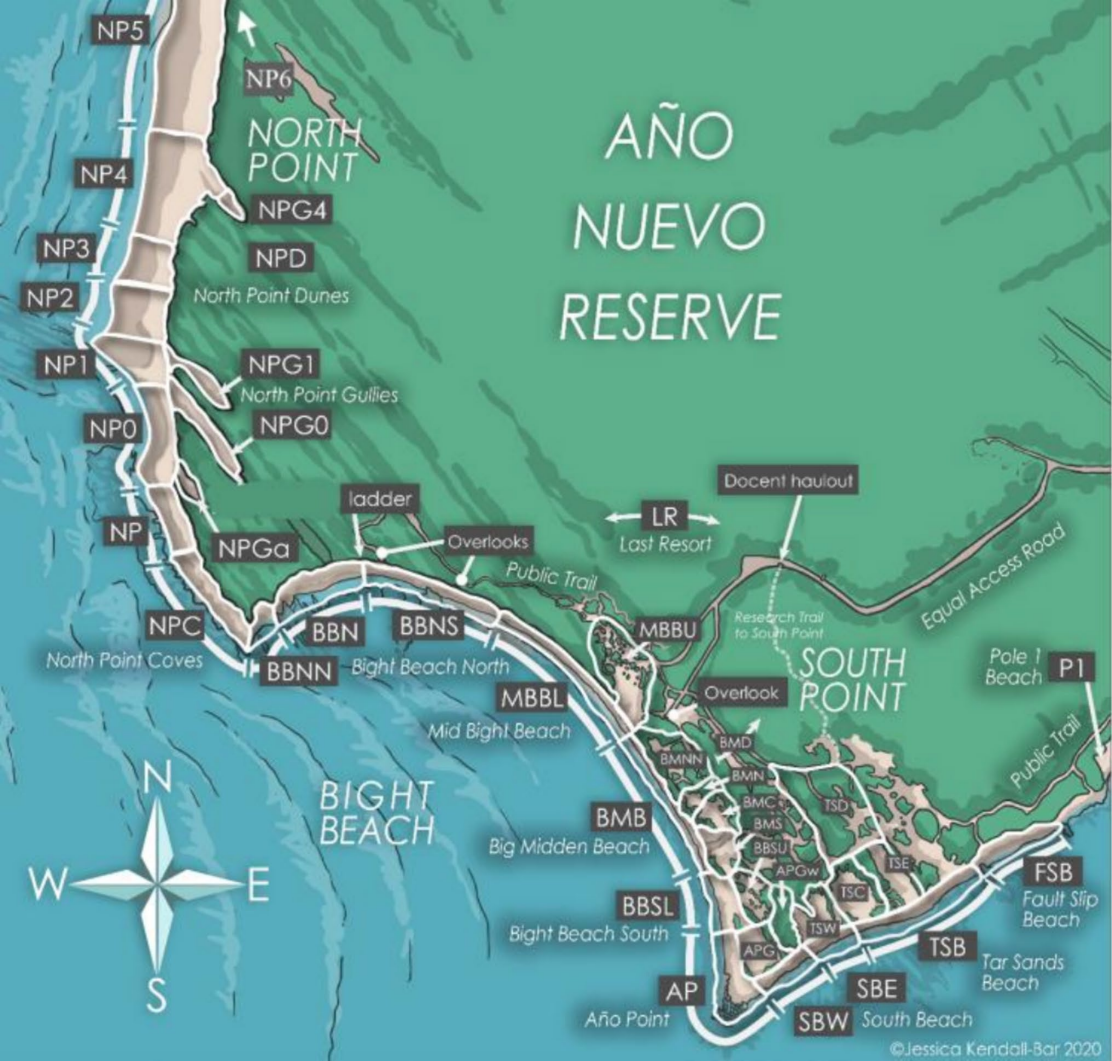
Illustration of potential pupping beaches within the Año Nuevo Reserve mainland. The named beach segments recorded in observations are used to identify fine-scale philopatry (Illustration by Jessica Kendall-Bar)

Data were collected from the northern elephant seal colony within Año Nuevo Reserve (37.12° N, 122.31° W) in San Mateo County, California. The colony encompasses the mainland and Año Nuevo Island, which is 500 m offshore (Le Boeuf 2011). Northern elephant seal pups were first observed on Año Nuevo Island in 1961, and the first pup on the mainland was observed in 1976 (Le Boeuf 2011; Orr and Poulter 1965). Between 1961 and 2015, the birth rates of the Año Nuevo mainland colony increased from an initial 12 to nearly 2000 pups annually (Lowry 2014). A long-term mark–recapture program at Año Nuevo began in 1967 (Le Boeuf et al. 2019), and the ongoing intensive mark–recapture efforts have generated robust sample sizes for numerous in-depth population dynamic and life-history studies (Beltran et al. 2021; Condit et al. 2022, 2023). This colony is unique as its history has been well documented since initial colonization (Le Boeuf 2011). The Año Nuevo mainland colony spans roughly 3219 m of beach (Holser et al. 2021). For clarity, and to provide detailed beach usage information, we separated the reserve into 38 different beaches (Fig. 1). We grouped these beaches into three regions differentiated by unique physical characteristics to analyze how straying may indicate the desired beach structure (Table 2; Supplemental material). The Northern region is defined as all beaches north of NPC, the Central consists of beaches MBBU, MBBL, BBN, and BBNS, and the Southern region of the colony includes all beaches southeast of BMB (Fig. 1).

### Data collection

The long-term mark–recapture program at Año Nuevo Reserve encompasses two separate fieldwork efforts: weanling weighing and flipper tag resights. Weanling weighing efforts occur between February and March, immediately after pups are weaned from their mothers. Researchers collect weight, length, and girth measurements from ~100–300 weanlings each season. In addition, each handled weanling receives two green flipper tags with a unique alphanumeric identification number that allows researchers to track individuals within and across colonies through mark–recapture efforts and integrate new individuals into the tracked population (Beltran et al. 2025).

Near-daily flipper tag resights occur at Año Nuevo between October and June, with ~360,000 observations of 50,000 seals recorded (R. Beltran, *pers. comm.*). Resight efforts are heightened during the breeding and molt seasons to account for higher abundances of seals returning to the colony at these times. Researchers document flipper tags and/or dye marks of individuals within the colony, along with the date, age, sex, molt status (during molt season), pup status/ID (during breeding season), and the beach where each individual was observed (Beltran et al. 2025). Resights are then entered into the elephant seal research program’s resight database which encompasses nearly 58 years. For this project we only analyzed data from the past 22 years (2001–2023) to maintain consistency of methods.

### Georeferencing

We georeferenced the beaches and gullies used for resight efforts to quantify distances between pupping events. Specifically, we utilized the freehand raster georeferencing plugin in QGIS to assign coordinate values to each beach using satellite imagery from Planet (PBC 2022). Coordinate values were acquired using the UTM zone 10N coordinate projection. Coordinates from the estimated center of each beach/gully were calculated using the georectified version of the map in Fig. 1. There were 13 beach names recorded in resight entries that do not exist in the current colony map primarily due to changes in beach names over time. Of these beach names, one was omitted, and we estimated the most appropriate coordinate values for the other 12 (Table 1).

**Table 1:** Beach names present in data but not present in Fig. 1 and the solutions for estimating their coordinates

Beach names	# Observed	Issue	Solution
NPG	5	Non-specific gully name	Coordinate values assigned to center NPG0 and NPG1 as these were the only named gullies at the time of resight
NP2/3	2	Non-specific beach name	Coordinate values assigned in between NP2 and NP3
MBB	11	Non-specific beach name	Coordinate values assigned in between MBBL and MBBU
BBS	18	Non-specific beach name	Coordinate values assigned in between BBSL and BBSU
BM	1	Non-specific beach name	Coordinates values assigned in between following cluster of possible sites: BMB, BMNN, BMD, BMN, BMC, BMS
SBEU	3	Too specific	Additional coordinate values attributed to SBE, in ‘upper’ region
SBEL	2	Too specific	Additional coordinate values attributed to SBE, in ‘lower’ region
TSBE	1	Too specific	Additional coordinate values attributed to TSB, in ‘eastern’ region
NNPG3	1	Typo	Given same coordinate values as NPG3
NPG2	1	Beach name changed	Coordinates based off 2005 image
NPG3	1	Beach name changed	Coordinates based off 2005 image
NPG2B	1	Beach name changed (last resight in 2013)	Coordinates based off 2005 image
APBN	1	Uninterpretable	Omitted

We used R statistical software to calculate distances between each possible combination of observed natal and pupping sites (R Core Team 2021). Spatial vector data was organized using the *sf* package (Pebesma and Bivand 2023; Pebesma 2018). Combining the 40 existing beaches and the 12 additional beaches not featured in Fig. 1, we created a 52 × 52 matrix to determine the straight-line distances between each possible beach combination. With this methodology, it is important to note that matrix values depict distances between the centroid of each beach seals were observed in, not the seal’s precise geographic position within the reserve (i.e., a beach that is 20 m long may contain two seals 19 m apart from each other, but their distance in the analysis would be the same). Due to this, distance values are discrete rather than continuous. Figures were created using Rstudio’s *ggplot2* package (Wickham 2016), and code utilized R package *tidyverse* (Wickham et al. 2019).

### Fine-scale natal site fidelity

This study only incorporates individuals from the mainland colony, which will be called the Año Nuevo colony hereafter. Data of all known mother–pup pairs were extracted from the resight database between 2000 and 2023, resulting in a total sample size of 124 mother–pup pairs, each with known birth locations. Fifty-five of these pairs included repeat mothers with different pups—as elephant seals only have one pup annually, repeat mother–pup pairs refer to records that document mothers returning for more than one breeding season. For a pair to qualify for data extraction, a mother’s birth beach (natal site) and her subsequent pupping beach must be known. Additionally, both beaches must be located within Año Nuevo Reserve. Accordingly, we discarded data pairs containing females immigrating from other colonies. If mother and pup were observed in different beaches throughout the same breeding season, the pupping site was defined as the first beach where the pup was observed.

**Fig. 2:**
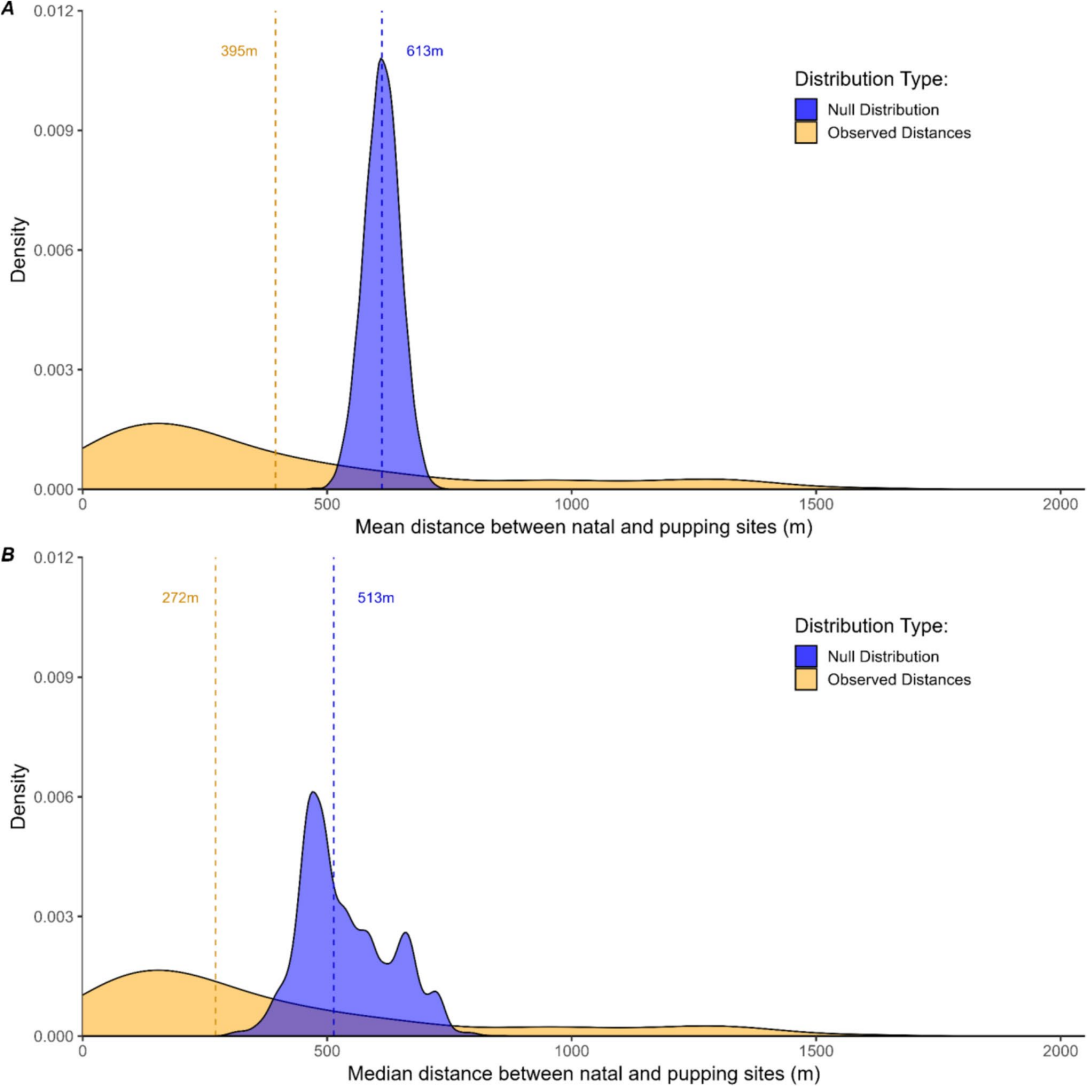
Observed distances between natal and pupping sites for *n* = 124 known mother–pup elephant seal pairs were closer than expected from random chance, indicating fine-scale philopatry in northern elephant seals. Density plots depict the frequency of geographic distances between mother and pup birth sites. The blue curve represents the null distribution of randomized mean (**A**) and median (**B**) distances for comparison. Dashed lines highlight the associated observed and null distances. *p *value <0.001 for the permutation test. Observed distances are statistically unimodal (dip test *p*-value = 0.09, R package *diptest* (Maechler 2021))

The beach name of a female’s birth site (natal site) and their subsequent pupping sites were paired based on individual mother and pup IDs. Distances between natal and pupping sites were assigned from the distance matrix described earlier based on the beach name for each mother–pup pair. We conducted a permutation test to evaluate our null hypothesis that our sample group’s mean distance between natal and pupping sites is not significantly different than that decided by random chance. Our alternative hypothesis was that the mean distance between natal and pupping sites of female seals at Año Nuevo is shorter than by random chance, indicating fine-scale philopatry. We randomized distances of the sample group by shuffling the mother/pup beach assignments, thus randomizing the possible distances between natal and pupping sites. We randomized the sample group 10,000 times and created a null distribution using the mean and median distances from each run (Fig. 2). *p *values were calculated by dividing the number of times the observed value appears as one of the null values by 10,000 (the total number of null values). We calculated *p*-values for both mean and median values.

**Fig. 3:**
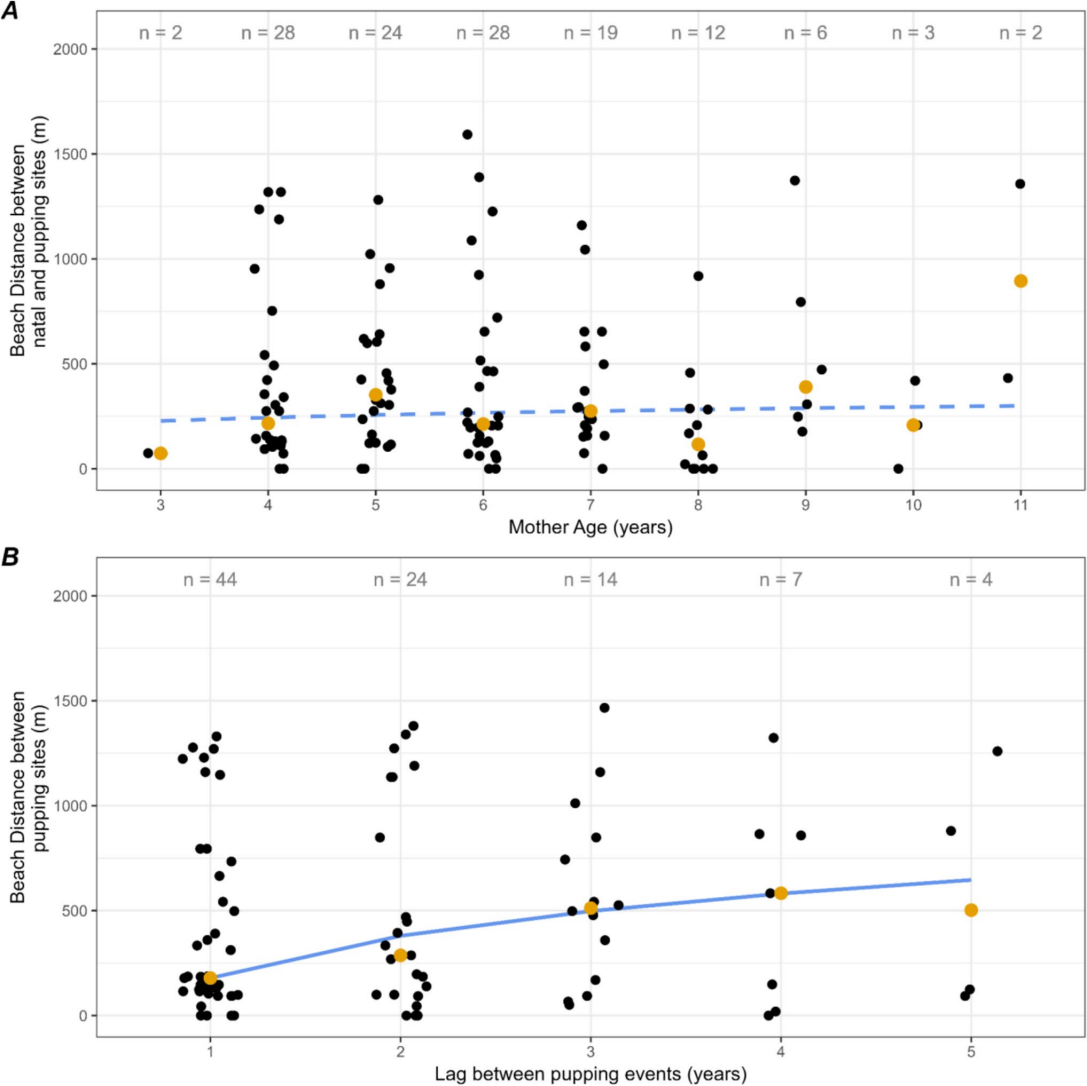
For *n* = 124 mother–pup elephant seal pairs, distance between natal and pupping sites was unrelated to maternal age but strongly associated with the number of years between pupping events. Both scatterplots depict the raw data distributions (black) and median (gold) of geographic distances. The sample size for each *x* variable is indicated as ‘*n*’. (**A**) The dashed model fit indicates no significant relationship between maternal age and distance between pupping site and natal site. (**B**) Distance between a mother’s subsequent pupping sites as a function of years between pupping events (i.e., lags), with a solid line indicating the significant relationship between lag year and distance

We also analyzed the distance between the mother’s natal site and each subsequent pupping site as a function of the mother’s age. We conducted a median one-tailed quantile regression analysis with the hypothesis that distance between the mother’s natal site and subsequent pupping site would increase with the mother’s age (Fig. 3A).

### Fine-scale site fidelity across breeding seasons

We calculated fine-scale site fidelity across breeding seasons by comparing distance between a mother’s first and subsequent pupping events. We isolated mothers recorded to have produced pups at least twice to guarantee presence in the colony during multiple breeding seasons so fine-scale site fidelity could be compared. Fifty-four of the 124 mother–pup pairs included mothers that pupped at least twice. These pairs consisted of the observation beach of the first pup and the observation beach of later pups, joined by mother ID. Respective distances were pulled from the distance matrix and applied to each pair. The same permutation test used in the ‘*Fine-scale natal site fidelity*’ section was used to develop a null distribution for distances between first pupping and later pupping.

We also analyzed distances between each mother’s pupping sites as a function of lag years between pairs of pupping events. While this analysis only includes mothers who gave birth at least twice, multiple pupping events are compared against each other (to calculate lag year between each breeding event) as opposed to being compared to the first pupping event (unique to the analysis described in the previous paragraph). This means the total data points for the lag year analysis are greater (*n* = 94) than the sustained site fidelity analysis (*n* = 54), although both analyses include the same number of individual momIDs (*n* = 30). We tested these relationships using a median quantile regression model, with a one-tailed approach to reflect the directional hypothesis that distance would increase with lag years (Fig. 2B). In the quantile regression model, we omitted a single value for lag year 6, as one value was not sufficiently representative of a lag year. Remaining lag years had a sample size of at least 3.

### Regional shifts in pupping location

We plotted the natal and subsequent pupping sites of 124 female elephant seals to determine if female elephant seals exhibited stronger philopatry in different colony regions. We defined three separate regions of the colony based on different qualitative topographic characteristics. The northern region has a more dynamic coastline which includes shifting sand dunes. The central region comprises relatively narrow beaches, with storm exposure and high tide periodically limiting beach space during the breeding season. The southern region includes a large sandy peninsula with a protected beach on the southern coast of the reserve. We used a multinomial regression (using the *multinom*() function from the *nnet* package in R) to quantify regional shifts in pupping location (e.g., to determine whether mothers born in the southern region of the colony were more likely to produce pups in the southern region, or in other regions). Pupping locations for both pups (response variable) and mothers (predictor variable) were categorical with three levels: North, Central, or South.

## Results

### Fine-scale natal site fidelity

We measured distances between natal and pupping sites of 124 adult female elephant seals, ranging in age from 3 to 11 years old. The mean distance between natal and pupping sites of adult females was 395 m (Fig. 2A), which is significantly shorter than the expected distance of 613 m if pupping sites were chosen at random (*p* < 0.001, Fig. 2A). Median values indicate even shorter distances, with the null observed median distance between natal site and pupping site being 513 m, and the observed median distance being 272 m (Fig. 2B). The maximum distance between natal and pupping sites was 1593 m. 25% (*n* = 32; Q1) of females gave birth within 124 m of their natal site, and 75% (*n* = 93; Q3) of females gave birth within 522 m of their natal site. 9% (*n* = 12) of females gave birth within their natal beach area (Fig. 2A).

Distances between natal and pupping sites were variable as a function of maternal age and there were no significant differences between age groups, although median distances seemed to increase slightly with age (Fig. 3A). Median distances remained below 500 m between ages 3 and 8 years old. Outliers (seals whose pupping distance was much further from their birth distance) were present in age groups 4, 6, 7, and 8.

### Fine-scale site fidelity across breeding seasons

**Fig. 4:**
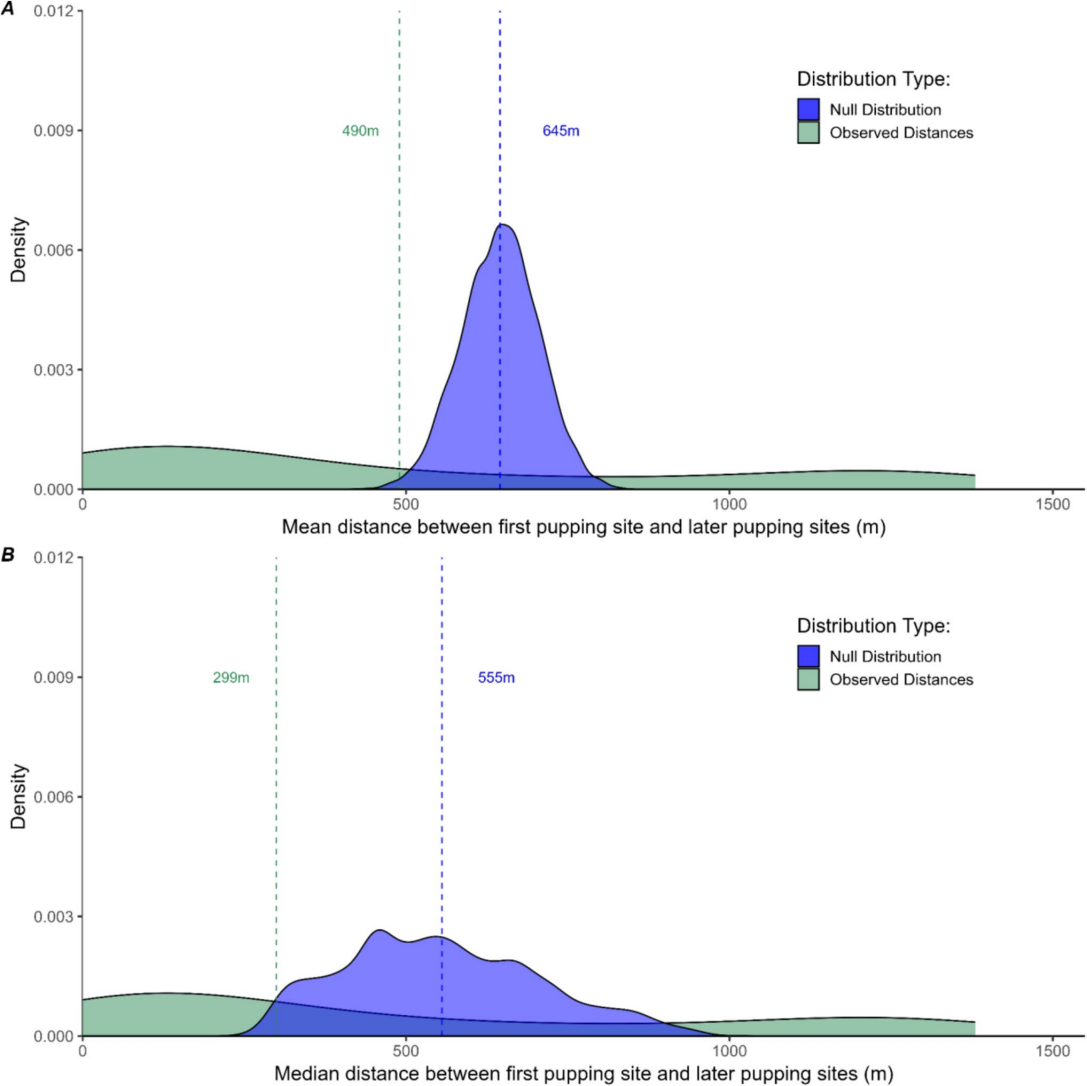
Observed distances between first and subsequent pupping sights were closer than expected from random chance, indicating northern elephant seals exhibit fine-scale birth fidelity. Density plot depicting the frequency of geographic distances between the site of first pupping and subsequent pupping (green) for *n* = 54 female seals. The null distribution curve, which represents 1000 randomized mean (**A**) and median (**B**) distances, is shown in blue for comparison. Data only incorporates known mother–pup pairs of mothers who gave birth at least twice. *p* value <0.003. Bimodality coefficient = 0.722 (R *mousetrap* package, Kieslich et al. 2019)

The mean distance between the first and later pupping sites was 490 m, which was less than the mean distance of the null distribution from the permutation test (645 m; Fig. 4). Median distance values representing this same analysis were skewed even shorter, with a median null value of 555 m, and the median observed value of 299 m. The observed density curve displayed a bimodal relationship, with the most frequent distance between the first and later pupping sites at 131 m. A slight increase in abundance peaked at 1204 m (Fig. 4). 27.78% (*n* = 15) of the females gave birth within 100 m of their first pupping site, while 74.07% (*n* = 40) gave birth within 861 m of their first pupping site. Comparison with the null distribution suggests the distance between the first and subsequent pupping sites was closer than expected from random chance (*p* < 0.003, Fig. 4).

Median distances between pupping sites increased significantly as a function of lag year (*p* = 0.0046; Fig. 3B). The distance between two pupping events for a single female was closer with a 1-year lag compared to longer lags.

### Regional shifts in recruitment

**Fig. 5:**
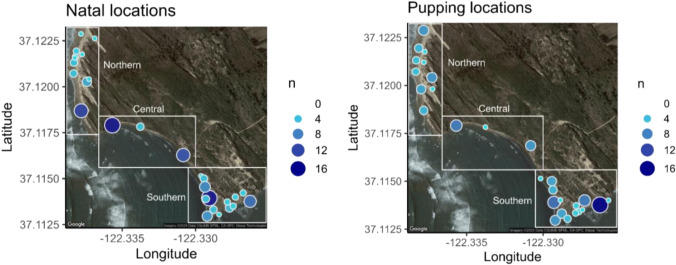
Map of natal and subsequent pupping site abundance of *n* = 119 adult female elephant seals. Año Nuevo is sectioned into three regions for fine-scale colony analysis: northern, central, and southern. Circle size and color represent the density of seals at each beach location. The beach location for P1 was excluded in this figure, requiring the omission of 5 mother and pup pairs. Beach P1 is considered a Southern region beach in numerical analysis

**Fig. 6:**
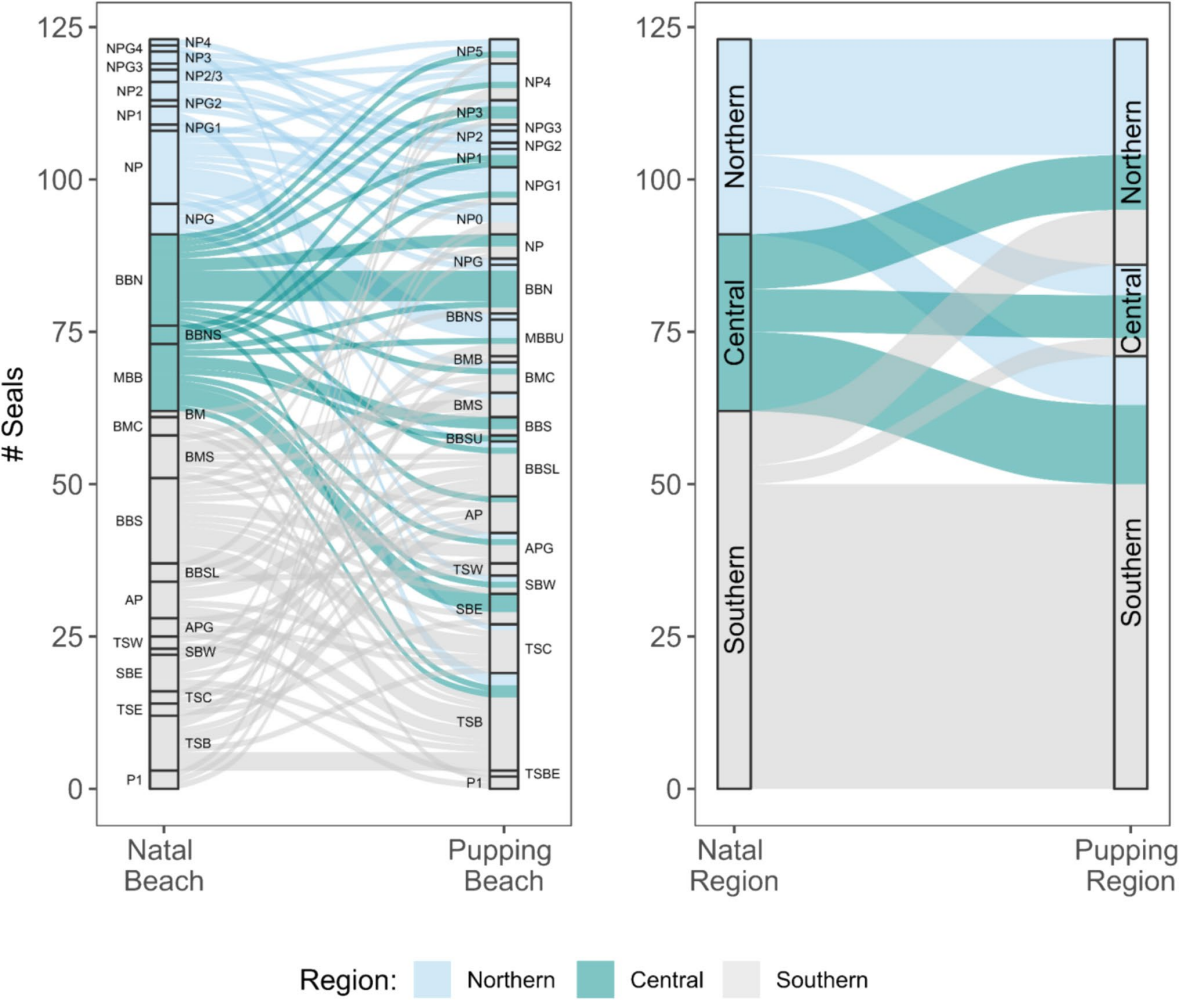
Alluvial plots depicting site fidelity and straying trends of Ano Nuevo’s northern elephant seal colony at a beach (left) and regional (right) resolution. Alluvial pathways connect the maternal seal’s natal site (left strata) and their subsequent pupping site (right strata). Pathway thickness reflects # of seals connecting each strata

We mapped natal (Fig. 5A) and later pupping (Fig. 5B) sites of 123 adult female seals ranging in age from 3–11 years old to explore spatial differences in philopatry Año Nuevo Reserve (Fig. 5). One mother–pup pair was excluded from this analysis due to the natal site not being present on our map renderings. Frequent natal sites included BBN (*n* = 15) and BBS (*n* = 15) (Fig. 5A). Only one pupping site (TSB; *n* = 16) was frequently used (Fig. 5B). Over 50% of mothers born in the southern and central regions produced pups in those same regions, but some mothers gave birth in other regions. Southern mothers were less likely to give birth to central pups (12.9 times less likely) or northern pups (4.3 times less likely) than to southern pups (*p* < 0.0001 for both; Fig. 6, Supplemental Table 1). Compared to producing southern pups, central mothers were 3.3 times less likely to produce central region pups (*p* = 0.0674; Fig. 6) but about equally likely (1.2 times less) to produce northern pups (*p* = 0.638). Compared to producing southern pups, northern mothers were 2.6 times more likely to produce northern region pups (*p* < 0.034; Fig. 6) and about equally likely to produce central region pups (2.3 times less likely; *p* = 0.22). The average beach-scale resolution in this study is 71.61 m, calculated from the average area (m^2^) of all beaches present in mother–pup analysis (Supplementary Table 2). Since beaches vary in size, this resolution is further summarized by region in the following sections.

#### Northern region

Of the seals born in the northern region, the greatest number were born at beach site NP. The number of females born at NP was greater than the number that returned to pup there. In contrast, neighboring beaches NP4, NP5, and NPG1 had more females pupping there than the number of females born. The remaining beach sites in the northern region remained relatively stable between natal and pupping numbers. The northern region experienced a more dispersed distribution in pupping sites compared to natal sites (Fig. 5), which were more localized in NP. While 51% of females that reproduced in the northern region were born there themselves (*n* = 19), 24.3% were born in the central region (*n* = 9) and another 24.3% were born in the southern region (*n* = 9) (Fig. 6). Overall, more seals gave birth and raised their pups in the northern region (*n* = 37, 30% of sample population) of Año Nuevo than were born there themselves (*n* = 32, 26% of sample population). An increase in pupping frequency and dispersal across the northern region of Año Nuevo is noteworthy as this region has experienced sand dune shifts over time, which could influence site selection. The fine-scale resolution of the beaches in the northern region is 83.61 m on average, with a minimum of 21.44 m and maximum of 142.89 m (Supplemental Table 2). Resolution is defined by straight-line distances of averaged beach areas (m^2^), as beaches vary in size.

**Table 2:** Natal and pupping sites of known mother–pup pairs compared in the context of three broader regions of Año Nuevo colony; Northern (includes all beaches north of NPC), Central (includes beaches between BBNN and MBBU), and Southern (includes all beaches south/east of BMB, including inland regions)

Region	Beaches	Natal (*n*)	Pupping (*n*)	Natal (%)	Pupping (%)
Northern	NPC, **NP**, NPGa, **NP0**, **NPG0**, **NPG1**, **NP1**, **NP2**, **NP3**, **NP4**, **NPG4**, NPD, **NP5**, NP6, ***NNPG3****, ****NP2/3****, ****NPG****, ****NPG2B****, ****NPG2****, ****NPG3***	32	37	26	30
Central	**BBN, BBNS**, **MBBU, MBBL,** BBNN, ***MBB***	29	15	23.6	12.2
Southern	**BMB**, BMNN, BMD, BMN, **BMC**, **BMS**, **BBSU**, APGw, **BBSL**, **AP**, **APG**, **TSW**, **SBW**, **SBE**, **TSC**, TSD, **TSE**, **TSB**, FSB, **P1**, ***BM*, *SBEU*, *SBEL*, *TSBE*, *BBS***	62	71	50.4	57.8

The Beaches column identifies all beaches that make up each region. Beach names in **bold** are those observed as a natal and/or pupping site and are plotted in Fig. 5. *Italicized* beach names were entered as an observed natal and/or pupping site, but do not exist in Fig. 1. Names of beaches are ordered from northernmost to southernmost. For more information on italicized beaches, see Table 1. Natal and Pupping % represents the proportion of the 123 mother/pup pairs with a natal/pupping site in each region.

#### Central region

Natal sites were highly clustered in BBN. The central region experienced a decline in females returning to their specific natal beach and broader natal region (central) to pup. 23.6% of seals in the sample population were born in this region but only 12.2% returned to pup (Table 2). While the majority of pupping females in the central region were born there, 33.3% of central females (*n* = 5) were born in the northern region and 20% (*n* = 3) were born in the southern region. This region was unique in that more seals born here pupped in the northern and southern regions than returned to pup in the central region (Fig. 6). The fine-scale resolution of the beaches in the central region is 71.26 m on average, with a minimum of 51.88 m and maximum of 100.89 m (Supplemental Table 2).

#### Southern region

Female natal sites were clustered at BBS, and pupping sites were highly aggregated in TSB. Sites BBS and TSB are 248 m apart and experienced contrasting abundance shifts. The relative abundance of natal sites at BBS (*n* = 15) was larger than the abundance of seals that used BBS to pup (*n* = 3). In contrast, the abundance of seals born at TSB (*n* = 9) was lower than that of seals that used TSB to pup (*n* = 16). 50.4% of the sample population was born in the southern region, while 57.8% of sample seals gave birth in this region (Table 2). Of the 71 seals that gave birth in the southern region, 70.4% were born in the southern region themselves, 18.3% were born in the central region, and 11.2% were born in the northern region. The fine-scale resolution of the beaches in the southern region is 61.71 m on average, with a minimum of 33.58 m and maximum of 81.52 m (Supplemental Table 2).

## Discussion

While broad-scale natal philopatry has been documented in northern elephant seals, we lack a fundamental understanding of whether females exhibit fine-scale (within-colony) natal philopatry and whether it persists over their lifetime (max = 21 years for females, although there is substantial variation and the mean lifespan of females is around 1 year old: Condit et al. 2013, Le Boeuf et al. 2019, Beltran et al. 2025). We utilized data obtained from extensive mark-recapture efforts over the past 23 years to analyze fine-scale natal philopatry and sustained site fidelity within the northern elephant seal colony at Año Nuevo Reserve, and to better understand straying patterns. We only analyzed females that returned to the Año Nuevo colony to give birth rather than the elephant seal population as a whole (i.e. individuals born at the colony but breed at a different colony are excluded from this study). Given this scope, ‘straying’ refers to fine-scale straying from the immediate natal site while remaining within the colony. We found that females give birth an average distance of 395 m and a median distance of 272 m from their natal site. Both distances are significantly shorter than expected by chance.

Fine-scale site fidelity in relation to the first pupping site was sustained over multiple pupping events, although the mean and median distances were further than those in relation to natal sites (mean = 490 m, 395 m, respectively). This distribution was bimodal, possibly as a result of the geographic features of Año Nuevo constraining birth sites. There was no significant correlation between distance of natal site and pupping site as a function of mother age, although median distances seem to increase slightly with age. Increased variability in older years may be due to decreased sample sizes at older ages (Fig. 3A). We found significant correlation with distances between pupping sites as a function of lag years between a mother’s pupping events (Fig. 3B). Natal straying (patterns associated with individuals that did not maintain natal philopatry) was most prominent in the colony’s central region. The northern and southern regions experienced an increase in pupping site abundance, with animals abandoning natal sites in the central region.

Evidence of sustained on-land site fidelity across breeding seasons (at varying scales) has been observed for numerous pinniped species, including northern fur seals (*Callorhinus ursinus*) (Hoffman and Forcada 2012), Galapagos sea lions (*Zalophus wollebaeki*) (Wolf and Trillmich 2007), gray seals (*Halichoerus grypus*) (Pomeroy et al. 2000, 2001), Weddell seals (*Leptonychotes weddellii*) (Cameron et al. 2007; Croxall and Hiby 1983), southern elephant seals (*Mirounga leonina*) (Fabiani et al. 2006; McMahon and Bradshaw 2004), and now northern elephant seals (*Mirounga angustirostris*). Southern elephant seals of the Falkland Islands have been observed pupping an average distance of 500 m from their previous pupping sites (Fabiani et al. 2006), and we observed nearly the same average distance for northern elephant seals at Año Nuevo (490 m, Fig. 4) in relation to their first pupping site. This similarity is noteworthy, considering the difference in colony site size. The Falkland Island colony is over 1000 m longer than Año Nuevo (Falkland Islands total beach length: 4354 m; Fabiani et al. 2006. Año Nuevo total mainland beach length: 3219 m; Holser et al. 2021), which suggests that colony site size does not increase the frequency of site fidelity.

Of the previously listed pinnipeds, only gray seals (Pomeroy et al. 2000), northern fur seals (Hoffman and Forcada 2012), and southern elephant seals (Fabiani et al. 2006; Hofmeyr et al. 2012; McMahon and Bradshaw 2004) exhibited significant fine-scale natal philopatry. While phocid species that breed on pack ice cannot exhibit philopatry due to the dynamic nature of their habitat, hooded and harp seals have been observed to breed in approximately the same areas in which they were born (Davis et al. 2008; Hammill and Stenson 2022). Northern elephant seals are known to exhibit cross-colony philopatry (Reiter et al. 1981; Zeno et al. 2021; Le Boeuf et al. 2019), with 87–90% of female seals born in the Año Nuevo colony returning to the colony to breed (Le Boeuf et al. 2019). Complete (100%) cross-colony philopatry would limit population expansion and genetic dispersal, removing the movement and connectivity that enabled the species to survive an extreme genetic bottleneck. However, our study is the first examination of within-colony natal site fidelity for this species. Our findings indicate that northern elephant seals exhibit fine-scale natal philopatry, with an average distance from natal sites being 390 m, 75% of females pupping within 522 m of their natal site, and 12 individuals pupping within their natal beach. This is significantly closer than observed in their southern counterparts (~6 km from natal sites; Hofmeyr et al. 2012). However, differences in analysis resolution could skew this comparison as Hofmeyr’s analysis resolution scale was ~120 km, while our resolution scale was ~3060 m. The resolution of natal philopatry in our study is limited to the beach where each seal was observed rather than the specific location of each seal. Our findings of fine-scale philopatry in northern elephant seals are congruent with related phocid species, but present a species-specific genetic risk.

High site fidelity and philopatry rates are theorized to ensure environmental familiarity with an individual’s natal site and facilitate finding mates for species that encompass a large range (Clutton-Brock and Lukas 2012). Researchers have also theorized that colonizing seals exhibiting site fidelity may exhibit interannual associations, possibly contributing to conflict reduction through social familiarity (Pomeroy et al. 2005). While northern elephant seals have successfully recovered from near extinction with this life history strategy, there are concerns for the future persistence of the population considering the resulting limited genetic diversity. Populations with low genetic variability are inherently at risk of disease and parasite transmission (Cross et al. 2009)—a risk which is further heightened in northern elephant seals as they congregate in high-density harems during the breeding season. Exhibiting high natal site fidelity can further limit genetic variability during their time on land by increasing inbreeding risk. However, avian species exhibiting extreme philopatry manage to avoid inbreeding, although the mechanism is unknown (Wheelwright and Mauck 1998). There is evidence that other phocid species that exhibit high natal philopatry can recognize their kin (Pomeroy et al. 2000), which may mitigate inbreeding within colonies, but this has not yet been documented in elephant seals.

We mapped the natal and pupping sites of 124 adult female seals to visualize patterns of natal straying within the Año Nuevo colony. Comparing the natal map with the pupping map (Fig. 5A, B), changes in numbers at a given site indicate straying. We found that the southern and northern regions increased in number while the central region decreased. Females born in the central region were 3.3 times less likely to pup in the central region, whereas they were equally likely (1.2 times less) to pup in the northern and southern regions (Fig. 6, Supplemental Table 1).

While most natal sites in the northern region were aggregated at beach site NP, pupping sites were more dispersed throughout the region (Fig. 5). The northern region of Año Nuevo is exposed to the effects of harsh winter storms, abrupt coastline changes, and shifting sand dunes. Changes in beach topography are reflected in satellite imagery between 2018 and 2023 (Supplemental material), revealing the expansion of gully NPGa following the record-breaking atmospheric river event that impacted the San Mateo coastline in late 2020 (US Department of Commerce 2021). Shifting dunes are thought to discourage fine-scale natal philopatry in gray seals on Sable Island—a species that otherwise exhibits fine-scale natal site fidelity (Weitzman et al. 2017). We hypothesized the dynamic nature of the northern region would increase straying towards the other regions. While there was more dispersal at a beach level (resolution; ~83.61 m, Supplementary Table 2), the northern region maintained a higher rate of natal philopatry than natal straying (resolution; ~1070 m). This suggests northern elephant seals may exhibit a level of plasticity in response to shifting dunes in the context of philopatry.

We identified the central region as the only region with a higher rate of outbound straying than inbound straying from other regions (Fig. 6). As the central region had the lowest abundance of both natal and pupping sites, respectively, this observed emigration towards higher-abundance harems corresponds with broad-scale emigration trends observed across colonies (Condit et al. 2023). Additionally, the central region has the least amount of available beach space amongst the three regions, potentially resulting in a smaller localized carrying capacity and further encouraging straying towards the two neighboring regions. A combination of these behavioral and environmental drivers may contribute to the region’s high rate of natal straying.

The southern region of Año Nuevo is the only region that maintained a high localized aggregation of natal sites and pupping sites, indicating a higher rate of natal philopatry than the central and northern regions. This region is characterized by stable beaches and dunes due to high vegetation density. The reliability of this region’s topographic structure may be a physical characteristic sought out by breeding females. The southern region still exhibits fine-scale straying, which could result from younger females getting shunted toward the outskirts of high-density harems by more dominant females during the breeding seasons, interfering with their ability to maintain site fidelity (Le Boeuf 2011).

Hypothesized drivers of fine-scale natal straying include topographic shifts and attraction towards higher-abundance groups within the harem. Additionally, founder fitness may affect the accuracy of fine-scale philopatry for different age demographics. Founder fitness refers to a higher survivorship of pups born to females that arrive at the colony early in the breeding season, as adult females can choose the most beneficial spot in the colony to rear their young (Pomeroy et al. 2001). Previous studies have indicated a survivorship advantage to founders’ fitness in gray seals (Pomeroy et al. 2001) and southern elephant seals (McMahon and Bradshaw 2004). In 1981, the researchers documented older northern elephant seals arriving earlier in the breeding season and exhibiting a higher weaning success rate (Reiter et al. 1981). While this supports the theory of founder’s fitness exhibited in northern elephant seals, the previous study noted that older females arriving late were aggressive enough to access prime pupping locations within the colony. This suggests older females may be able to maintain fine-scale philopatry more accurately than younger females regardless of arrival date; however, mother age in our study did not have a significant correlation with natal philopatry (Fig. 3A). Variation in reproductive success may also influence site selection. For example, a female who loses a pup before weaning may be more likely to stray, although data to test this phenomenon has not yet been collected. Examining pupping and weaning success in prior and future years with pupping locations and associated habitat characteristics could address this question.

Investigating distances between pupping sites between subsequent breeding events offers new insight into the processes shaping elephant seal site selection. We found that the distance between successive pupping sites increased with time between pupping events. Lags of more than 1 year (e.g., pupping events in years *t* and *t* + 2) could include either pupping events or no pupping events in the intervening year(s) (e.g., year *t* + 1); the relative frequency and role of each of those events is an important topic of future study. Intermittent pupping (i.e., years of skipped breeding or pupping) is an important life-history strategy for female elephant seals as it allows them additional time to replenish the fat reserves needed to wean healthy pups (Desprez et al. 2018). The observed correlation between lag year and increased breeding-site displacement could reflect fidelity to a mother’s previous breeding site more strongly than that of their natal or first pupping site. Fidelity to a mother’s previous breeding site has been documented in other colonizing species, such as blue-footed boobies (Kim et al. 2007). As our study only includes site shifts following successful birth events, our results do not capture potential effects of unsucessful birth events on subsequent site choice, which has been cited as a factor in site selection in other female-philopatric species (Öst et al. 2011). Ultimately, the drivers of fine-scale site selection are likely multifaceted, involving both physical condition and behavioral decision-making.

In conclusion, our study demonstrates high rates of fine-scale site fidelity in northern elephant seals, both to natal sites and to sites of first pupping, with fidelity being stronger to natal sites. We also find evidence of movement toward higher-density groups at a fine scale, consistent with patterns observed at broader cross-colony scales. Additionally, the distance between successive pupping sites increases with the number of breeding seasons between pupping events, suggesting that extended absences may influence site selection. Future research should examine environmental and behavioral drivers of site choice, and specifically compare site shifts following successful weaning versus pup mortality events to assess whether reproductive failure plays a role in subsequent breeding-site decisions. Investigating whether fine-scale patterns mirror broad-scale trends will also be valuable for understanding genetic dispersal and forecasting population growth and connectivity within and among northern elephant seal colonies.

## Supplementary Information

Below is the link to the electronic supplementary material.

Supplementary file1 (PDF 4869 KB)

Supplementary file2 (CSV 19864 KB)

## Data Availability

Data is available upon request.
