# Supplementary material for: Like mother like daughter: northern elephant seals exhibit fine-scale philopatry
Source: Oecologia. 2025 Dec 10;208(1):13. doi: 10.1007/s00442-025-05846-6 (PMC12695955; doi:10.1007/s00442-025-05846-6)
Supplement: Supplementary file 1 — Supplementary file1 (PDF 4869 KB) [file 442_2025_5846_MOESM1_ESM.pdf]

Like mother like daughter: Northern elephant seals exhibit fine-scale philopatry - Electronic Supplementary Material (ESM)

Isabella G. P. Garfield[1], Danial G. Palance[1], Max F. Czapanskiy[1], Daniel P. Costa[1,2], Roxanne S. Beltran[1]

[1] Department of Ecology and Evolutionary Biology, University of California Santa Cruz, 130 McAllister Way, Santa Cruz, CA 95060 USA

[2] Institute of Marine Sciences, University of California Santa Cruz, 115 McAllister Way, Santa Cruz, CA 95060 USA

Handling author email: Isabellagarfield@gmail.com

Declaration of Authorship

IG formulated the idea, conceived and designed the experiment, drafted the manuscript and coded figures. DP, MC, and RB mentored, provided feedback, guided analysis and provided valuable edits to manuscript and code. DC provided edits to the manuscript. DC and RB have both led elephant seal research at UCSC, spearheading the data collection used in this study.

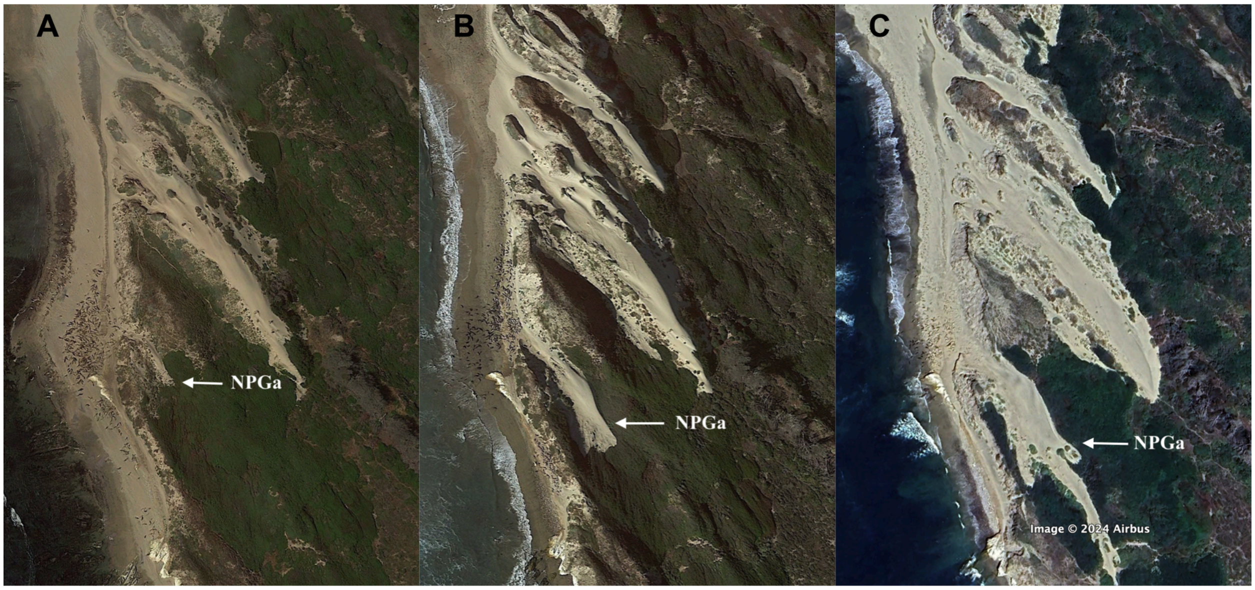

Supplementary Figure 1. Satellite images of Año Nuevo Reserve's northern region (elevation 26 ft, eye altitude 1642). Image A was taken on 5/9/2018 (Google Earth Pro, 2018). Image B was taken 9/27/2021 (Google Earth Pro, 2021). Image C was taken on 9/17/2023 (Airbus, 2024). Images ordered sequentially show the expansion of gully NPGa over time.

Supplemental Table 1. Fitted model from a multinomial logistic regression model predicting pup natal region as a function of maternal natal region, fit without an intercept (i.e., separate log-odds estimated for each mom birth region relative to the baseline pup region "southern"). Coefficients represent the log-odds of a pup being born in the specified region given the maternal origin, allowing for direct comparison across maternal source regions. Model was fit using the *multinom()* function from the *nnet* package in R (Venables, 2002).

**Pup Region**	**Mom Region**	**Estimate**	**SE**	**Z-value**	**P-value**
Central	Southern	-2.56	0.599	-4.28	1.86E-05
Central	Central	-1.2	0.658	-1.83	0.0674
Central	Northern	-0.847	0.69	-1.23	0.22

Northern	Southern	-1.47	0.37	-3.97	7.33E-05
Northern	Central	-0.223	0.474	-0.47	0.638
Northern	Northern	0.944	0.445	2.12	0.034

Supplemental Table 2. Summary of beach areas as calculated in ArcGIS. Straight line distances are calculated by taking the square root of the average, minimum, and maximum beach areas respectively.

Region	Average beach area (m^2)	Average straight line distance (m)	Minimum area (m^2)	Minimum straight line distance (m)	Maximum area (m^2)	Maximum straight line distance (m)
Northern	6,995.1	83.61	459.47	21.44	20,416.2	142.89
Central	5,078.5	71.26	2,691.36	51.88	10,179.76	100.89
Southern	3,808.3	61.71	1,127.37	33.58	6,645.67	81.52
Total	5127.47	71.61				
